# Feasibility of the SINEX program for patients with traumatic anterior shoulder instability

**DOI:** 10.1186/s40814-020-00679-x

**Published:** 2020-10-06

**Authors:** Amalie Nilssen Hagesæter, Tonje Løvold, Birgit Juul-Kristensen, Jesper Blomquist, Randi Hole, Henrik Eshoj, Liv Heide Magnussen

**Affiliations:** 1 Department of Health and Functioning, Western Norway University of Applied Sciences, Bergen, Norway; 2 Department of Sports Science and Clinical Biomechanics, University of Southern Denmark, Odense, Denmark; 3 Department of Orthopedic Surgery, Haraldsplass Deaconess Hospital, Bergen, Norway; 4 Department of Clinical Medicine, University of Bergen, Bergen, Norway; 5 Orthopedic Clinic, Haukeland University Hospital, Bergen, Norway; 6 Quality of Life Research Center, Department of Haematology, Odense University Hospital, Odense, Denmark

**Keywords:** Shoulder instability, Neuromuscular exercise, Feasibility studies

## Abstract

**Background:**

An optimal treatment for traumatic anterior shoulder instability (TASI) remains to be identified. A shoulder instability neuromuscular exercise (SINEX) program has been designed for patients with TASI, but has not yet been tested in patients eligible for surgery. The purpose of this study was to investigate and evaluate the feasibility and safety of the SINEX program for patients diagnosed with TASI and eligible for surgery.

**Methods:**

A feasibility study with an experimental, longitudinal design using both quantitative and qualitative research methods. A total of seven participants underwent the SINEX program, a 12-week exercise program including physiotherapist-supervised sessions. Feasibility data on recruitment, retention, compliance, acceptability and safety was collected through observation and individual semi-structured interviews. Clinical tests and self-report questionnaires were completed at baseline and 12 weeks follow-up. Clinical assessments included apprehension and relocation tests, shoulder joint position sense (SJPS), shoulder sensorimotor control measured by center of pressure path length (COPL) on a force platform, isometric strength measured by Constant Score-Isometric Maximal Voluntary Contraction (CS-iMVC), self-report questionnaires including Western Ontario Shoulder Instability Index (WOSI), Tampa Scale of Kinesiophobia (TSK) and Global Perceived Effect questionnaire (GPE).

**Results:**

With one participant recruited every 2 weeks, the recruitment rate was 50% lower than expected. Two of seven participants achieved compliance, defined as at least 66% completion of the scheduled home exercises and at least 50% attendance for the physiotherapist supervised sessions. Barriers for successful compliance were (1) inability to take along exercise equipment when travelling, (2) sick leave, (3) holidays and (4) lack of time/busy days. Four adverse events occurred, one of which was related to the intervention (patellar redislocation). All participants expressed satisfaction with the intervention and felt safe during the exercises. All participants improved in the GPE. Change greater than minimal detectable change (MDC) was reported in four participants in some of the outcome assessments. One of the seven participants declined surgery.

**Conclusion:**

Further assessment is required on several areas before performing an RCT evaluating the efficacy of the SINEX program for patients with TASI considered eligible for surgery. No adverse events suggest that the program is safe, but patients with general hypermobility may need additional adjustments to prevent adverse events in other areas of the body.

**Trial registration:**

ClinicalTrials.gov: NCT04152304, retrospectively registered

## Key messages regarding feasibility

- Neuromuscular exercises (The SINEX program) is deemed effective on traumatic anterior shoulder dislocation (TASI) in patients not referred to stabilization surgery. Evidence of the SINEX program on the same patients referred to surgery is unknown
- The SINEX program is feasible with a few adjustments in patients with TASI eligible for surgery. Home exercise options without equipment and exercises simulating real-case scenarios may improve feasibility
- Recruitment strategies need to be optimized in a future RCT. The SINEX program may be improved with a few adjustments

## Background

Shoulder dislocation seems to be a common problem among the young and active population. Liavaag et al. [1] found that the incidence of shoulder dislocations in Oslo, Norway, was 26.2 per 100,000 person-years. Ninety-five percent of all dislocations are anterior [2, 3], and traumatic shoulder dislocations can lead to shoulder instability. Traumatic anterior shoulder instability (TASI) may cause pain, loss of shoulder function and recurrent subluxation or dislocation [4]. Pain and loss of function may negatively affect most physical activities during work and leisure time, resulting in reduced shoulder-related quality of life [5]. In Norway, it is estimated that 25–40% of patients with a shoulder dislocation are treated surgically, 83% of which are anterior stabilization procedures [6].

The optimal treatment strategy for this patient group is unclear [7], and further challenged by the lack of knowledge on the natural course of shoulder instability [8]. Systematic reviews have reported surgery to reduce redislocations compared with non-surgical treatment for young athletic male patients with first time traumatic anterior shoulder dislocation [9, 10]. First-time shoulder dislocations are most often treated non-operatively, but there is a growing trend to treat high-risk (e.g. contact athletes) and younger patients (< 30 years) with initial surgical shoulder stabilization [11]. Nonetheless, whether this is the optimal strategy for *all* patients is still widely discussed [9, 10, 12]. Additionally, the evidence for prescribing non-surgical treatment using exercise intervention is limited [11], and more research is required to find the optimal treatment strategy for this patient group in order to further develop evidence-based practice [5, 6, 9, 11–14].

Neuromuscular exercises have shown to effectively reduce joint pain and improve function in other musculoskeletal conditions than the shoulder, and may lead to improved quality of life [15–17]. In patients with TASI, a proper exercise program also seems important to restore stability, and thus improve function [11]. Currently, only the Watson exercise program incorporating neuromuscular exercises has been tested in patients with *multidirectional* shoulder instability [18, 19].

A customized neuromuscular exercise program comprised of 12 weeks of training has recently been developed for patients with TASI with a maximum of five anterior shoulder joint dislocations [20]. The program is currently being investigated for safety and efficacy in TASI patients not eligible for surgery [20], but the program has not yet been tested in TASI patients eligible for surgery. Patients eligible for surgery were expected to have increased instability compared with patients not eligible for surgery. Therefore, the purpose of the present study was to evaluate the safety and feasibility of the SINEX program in patients diagnosed with TASI eligible for surgery.

## Methods

### Design

Our research was designed as a feasibility study without a control group [21, 22], using both qualitative and quantitative approaches. Hypothesis testing is inapplicable in a feasibility study [22]. The design is experimental and longitudinal, and our participants filled in questionnaires and were clinically tested before and after the intervention. At the end of the intervention, individual semi-structured interviews were conducted [21]. The current feasibility study design did not require blinding of participants or researchers [22]. AHN and TL were responsible for the physiotherapy treatment, testing procedures, and interviews. All eligible participants signed an informed consent form before baseline testing.

The project was approved by The Regional Committee for Medical Research Ethics: 2017/1189/REK vest.

### Participants and recruitment

Participants were recruited from two hospitals in Norway between September 2017 and January 2018. The original recruitment goal was a total of 20 participants. Since we conducted a feasibility study, a formal sample size calculation was not required [21]. The inclusion criteria were men and women aged between 16 and 45 years with minimum one traumatic anterior shoulder dislocation, who have been diagnosed with TASI and considered eligible for stabilizing Bankart surgery. Exclusion criteria were complex shoulder injuries not suitable for a Bankart procedure as determined by an orthopaedic surgeon, insufficient Norwegian language skills, and/or not being able to participate in a supervised exercise program.

### Intervention

The intervention was conducted according to the SINEX program, and a physiotherapist supervised a 12-week neuromuscular training program for participants with TASI [20]. The program includes seven exercises with seven progression levels ranging from basic to elite levels (A–G), individually tailored to each patient with regard to their respective shoulder function. Exercises at basic level are recommended to be performed daily and involve low resistance (2 × 20–25 repetition maximum [RM]), while exercises at the elite level are recommended to be performed three times weekly and include greater resistance (2 × 8–12 RM). The program includes exercises for both the glenohumeral and scapular muscles, as well as functional kinetic chain exercises. The full program with exercise descriptions and photos is provided in Additional file 1.

The participants were given the full SINEX program, with descriptions and photos of each exercise and all progression levels, and they were encouraged to progress the exercises themselves. During the weekly supervised sessions, AHN and TL assessed the quality of exercise performance. Satisfactory compliance was defined as ≥ 50% attendance of the supervised physiotherapy sessions and completion of ≥ 66% of the scheduled home exercises [20]. To monitor this, the participants received an exercise diary to document home exercise compliance and to record symptoms during their training. Participants were asked not to seek other treatment during the intervention.

To manage possible shoulder symptoms during the exercises, the participants used a pain and instability symptom scale ranging from 0 (no symptoms) to 10 (worst possible symptoms). If participants experienced symptoms greater than 5 during an exercise, they were instructed to adjust the exercise by reducing load or range of motion to prevent worsening of their symptoms. The participants were further informed about the difference between inflammatory symptoms (not acceptable during or after an exercise) and muscle soreness (acceptable). If participants experienced persistent worsening of their shoulder symptoms or a shoulder redislocation, they were immediately referred to the orthopaedic surgeon for reassessment of their shoulder.

### Feasibility evaluation

Five areas of feasibility were evaluated: recruitment, retention, compliance, acceptability and safety [22]. Feasibility was registered by continuous observation and note-taking during the clinical testing, the supervised sessions and the semi-structured interviews. The safety of the program was evaluated by registration of adverse events. Delayed-onset muscle soreness was expected and therefore not considered an adverse event. Quantitative and qualitative research questions regarding feasibility are presented in Table 1.

**Table 1:** Feasibility questions

Objective	Quantitative question	Qualitative question
**Recruitment**	◦ How many participants were recruited each week? ◦ How long did it take to recruit the required sample?	◦ Why did eligibles decide to participate? ◦ If the required sample size was not reached, what could have been done differently? ◦ What are the barriers to successful recruitment within the research sites?
**Retention**	◦ How many of the study participants remained in the study as they moved through the intervention? ◦ At what point did drop-out occur?	◦ Why did participants decide to withdraw from the study? ◦ What are the barriers to successful retention?
**Compliance**	◦ How many of the participants got the full “dose” of the intervention? ◦ Were there particular components for which compliance was especially low?	◦ Why did participants not adhere to the intervention protocol (or not adhere to particular components)? ◦ What are the barriers to successful compliance?
**Acceptability**	◦ How satisfied were participants with the intervention? ◦ To what extent did participants feel overburdened by data collection demands?	◦ What did participants like and dislike about the intervention? ◦ What changes to the intervention protocol would make it more acceptable? ◦ What did participants most dislike about research-related aspects?
**Safety**	◦ How many adverse events occurred during the project? ◦ How many adverse events were related to testing or intervention? ◦ How many participants felt safe or unsafe during the project? ◦ Do the physiotherapists responsible for administering the intervention believe the design of the intervention protocol to be safe?	◦ What adverse events did occur? ◦ What made participants feel safe or unsafe during the project? ◦ What could have further prevented adverse events? ◦ What changes could have been made to further increase the safety of the intervention according to the administering physiotherapists?

Inspired by Polit & Beck [22]. Process and safety-related questions used to collect data on feasibility

### Outcome assessments

#### Baseline characteristics

Information about gender, age, occupation, injured shoulder (right/left), injury in the opposite shoulder, dominant arm (right/left), shoulder dislocation debut (year), total number of shoulder dislocations and time since last experienced shoulder dislocation was collected.

#### Patient-reported outcome measures

##### Primary outcome measure

The Western Ontario Shoulder Instability Index (WOSI) is a questionnaire measuring shoulder-related quality of life for participants with shoulder instability [23]. WOSI has been found valid and sensitive to change and has shown excellent test-retest reliability for this patient group [23]. WOSI is the recommended patient-reported outcome for this patient group as it is more sensitive to change compared with other similar patient-reported outcome measures [23]. WOSI consists of a total of 21 questions and covers four domains: physical function sports/recreation/work, lifestyle and emotional well-being. Each question is scored on a visual scale ranging from 0 (best) to 100, with a total sum score of 2100 (worst). WOSI has been translated into Norwegian and validated in Norwegian shoulder instability participants with a minimal detectable change (MDC) of 339 WOSI points [24].

##### Seondary outcome measures

The Tampa Scale of Kinesiophobia (TSK) measures fear of movement and re-injury in people with musculoskeletal disorders and/or pain [25]. TSK consists of 13 items with a four-level ordinal scale (1 = strongly disagree to 4 = strongly agree). A score > 37 out of 52 suggests increased fear of movement and re-injury [26]. TSK has shown high test-retest reliability in participants with shoulder pain [25], and an MDC of 5.6 points has been established in participants with chronic pain [27]. TSK is translated into Norwegian, and acceptable validity and test-retest reliability has been demonstrated in participants with sciatica, but responsiveness was low to moderate [28].

A Global Perceived Effect (GPE) questionnaire was used to measure self-reported impression of change and treatment satisfaction with the SINEX program at post-testing [29]. Perceived change was self-reported on a seven-level ordinal scale (1 = completely recovered to 7 = worse than ever). Treatment satisfaction was self-reported on a five-level ordinal scale (1 = satisfied to 5 = dissatisfied) [29].

##### Clinical outcome measures

The apprehension test assesses anterior glenohumeral instability. The test has shown satisfactory inter-tester reliability, with acceptable sensitivity and specificity to detect anterior instability [30, 31]. A relocation test was also used to assess anterior glenohumeral instability, and although the test has good specificity [30], it is less reliable between examiners [32].

Joint position sense of the shoulder (SJPS) was measured with a laser pointer according to a previously validated protocol [33]. The test was performed blindfolded at approximately 90° shoulder flexion, and the difference between the reference point and the reposition point was calculated using simple trigonometry. This test has shown excellent test-retest and inter-tester reliability for low ranges of shoulder flexion (45–65°), but poor reliability in mid and high ranges (80–135°) [33]. An MDC of 3° has been established in a healthy population [33].

Shoulder proprioception and stability was further measured with center of pressure path length (COPL) using a Kistler 9286BA force platform [34]. COPL has shown acceptable test-retest reliability in a healthy population [34]. The patient was positioned in a prone position with lower extremities supported by a bench, and arms in extended positions with hands placed underneath the shoulder joints on the force platform. Three tests were performed and repeated three times each. Test 1 was with both arms on the force platform and eyes closed, test 2 was with the non-injured arm only on the force platform and eyes open, and test 3 was with the injured arm only on the platform and eyes open. Each test lasted 30 s with a 30-s pause between each attempt. The MDC for this test has been established to be 95.8 mm for both arms in a healthy population, 108.9 mm for the dominant arm, and 78.4 mm for the non-dominant arm [34]. In the present study, the MDC for the dominant arm was applied to the participant’s non-injured shoulder, and the MDC for the non-dominant arm was applied to the injured shoulder.

Isometric maximal voluntary strength in 90° shoulder abduction was tested in the scapular plane according to the protocol from the Constant Score (CS-iMVC) described by Moeller et al. [35] using an MIE Digital Analyser dynamometer strapped around the wrist. The protocol has shown acceptable test-retest and inter-tester reliability, and construct validity was confirmed in a population with shoulder impingement [35]. MDC is not reported for CS-iMVC, but based on traditional strength training principles, an increase of 30–40% on 1RM has been reported to correspond to real change [36].

### Data collection

The participants responded to WOSI and TSK on paper with the examiners present in the room. Baseline data were then collected, followed by physical testing. The order of the physical tests was identical for each participant, and the same examiner performed the same tests on all participants to minimize inter-tester variability. At post-testing, the patients also completed the GPE questionnaire [37].

After post-testing, a semi-structured interview took place. Each interview (lasting about 30 min) was conducted by the physiotherapist responsible for training the patient. An interview guide was developed based on the questions in Table 1. Examples of questions were as follows: *What is your overall experience of participating in the project? How would you evaluate the training and the testing? Were you at any time worried about your shoulder? Did you feel safe or unsafe at any point, and what contributed to said feeling? Do you still want to undergo surgery?* Other topics raised by the patient were followed up during the interview. Each interview was audio-recorded, transcribed and analysed using systematic text condensation [38]. When possible, the interview was conducted even if the patient did not complete post-testing.

## Results

Seven participants were included, with age ranging from 19 to 43 years old. Detailed baseline characteristics of all participants are presented in Table 2.

**Table 2:** Characteristics of participants at baseline (*n* = 7)

Characteristics				Participants			
Participant	1^a^	2	3	4^a^	5	6	7
Gender	F	M	F	M	M	M	F
Age	24	43	19	34	40	40	21
Occupation	S	OW	S	OW	OW	OW	PL
Shoulder dislocation (first time, year)	2016	2013	2016	2001	2010	1995	2013
Shoulder dislocation (total numbers)	2	7	3	8	5	4	1
Shoulder dislocation (since last, months)	2	24	1	1	2	2	60
Injured shoulder	R	R	L	R	R	L	R
Dominant arm	R	R	R	L	L	R	R
Injury in the other shoulder	No	No	No	No	No	No	No

*F* female, *M* male, *S* student, *O* office worker, *PL* physical labor, *R* right, *L* left

^a^Drop-out

### Feasibility evaluation

#### Recruitment

A total of 15 patients were referred to the researchers by the two orthopaedic surgeons responsible for recruiting, but only seven patients were included in the study. Over a 5-month period this corresponds to one participant in 2 weeks and was 50% lower than expected. Initially, eligible patients were informed of this project and encouraged to contact the researchers of the project themselves. After approval from the ethical board, this was changed, and the surgeons conveyed the contact info of eligible participants to the researchers, who could then contact potential patients directly. Before this change, several of the patients never called back to the researchers. After the approval from the ethical board, researchers were able to directly contact eligible patients. Two of those declined participation, and one was excluded because of insufficient language skills. Why two patients refused to participate is not known, as patients may according to the informed consent refuse participation without providing a specific reason. The most common reasons why patients choose to participate in the project were either to increase their chance of successful surgery, to eliminate the need for surgery, or because their orthopaedic surgeon had recommended physiotherapy.

#### Retention

Four out of seven participants completed the 12 weeks of intervention. Two participants dropped out (participant 1 at week 2, and participant 4 at week 8). For participant 1, the reason was unknown (lost-to-follow-up), whereas participant 4 discontinued due to a fall during recreational activities that resulted in a shoulder redislocation and subsequent stabilizing surgery. Participant 1 completed neither the post-testing nor the qualitative interview. Finally, participant 2 only completed 8 weeks of the intervention due to a previously scheduled surgery that the participant was not willing to postpone, despite participation in the study.

#### Compliance

Only two participants (3 and 6) achieved full compliance with the intervention (physiotherapist sessions and home exercises). Six participants [2–7] achieved full compliance with the physiotherapist supervised sessions. There was no difference in demographic characteristics between those who adhered and those who did not. In the interviews, participants revealed barriers regarding successful compliance to be the following: (1) inability to take along exercise equipment when travelling (for example the fitness ball), (2) sickness, (3) holidays and (4) lack of time. One participant suggested that exercises not requiring equipment would make it easier to carry out the program during travels.

#### Acceptability

All six participants expressed satisfaction with participating in the study, and they did not feel overburdened by data collection. A few participants revealed difficulties relating to the questions in the WOSI and TSK questionnaires. For example, the fact that WOSI uses a recall period of only 7 days was irrelevant to some participants, as they had previously given up certain sports or similar activities. All participants were positive about the opportunity for exercise progression, and some mentioned that they enjoyed the idea of being able to progress the exercises themselves. The participants appreciated the weekly individual physiotherapist supervised sessions, and one of them wished there had been more supervised sessions.

#### Safety

Four adverse events occurred during the project period: two shoulder redislocations, one lumbar disc herniation and one patellar redislocation. Both shoulder redislocations took place during sports activities outside the intervention program, and the herniation occurred when the participant lay in bed. The patellar dislocation was the only adverse event related to the intervention itself and occurred while performing exercise 4A (participant placed in a prone position with legs on a fitness ball). The participant was previously diagnosed with hypermobile joints and had dislocated his/her patella several times before. Several participants reported muscle soreness, which was expected and not defined as an adverse event. None of the participants reported pain or discomfort in the injured shoulder during the intervention or clinical testing. All six participants felt safe during the intervention and clinical testing, despite the fact that they were worried about unexpected movements, such as falling, in everyday life. Participants expressed that the weekly physiotherapist supervised sessions and the symptom scale contributed to feeling safe while exercising.

### Outcome assessments

Overall, the outcomes demonstrated few significant changes following the intervention (Table 3). Only participant 6 achieved change greater than the MDC for WOSI. No change was registered in any of the participants for the TSK, apprehension and relocation tests, or the CS-iMVC. However, changes greater than the MDC were discovered in SJPS (participant 7), and in testing the injured shoulder with eyes open, test 3, on the force platform (participants 2 and 5). There was no clear link between outcome improvements and intervention compliance. All participants reported some/much improvement on the GPE. One of the seven participants chose to decline surgery.

**Table 3:** Outcomes at pre- (T0) and post- (T1) testing (*n* = 7)

Outcomes	Participants								
		1*	2	3	4*	5	6	7	MDC
**WOSI** (0–2100) *Change score*	T0 T1	1052 – –	157 144 13	546 – –	1044 – –	493 302 191	**671** **9** **662**	1338 1053 285	339
**TSK** (0–52) *Change score*	T0 T1	33 – –	30 32 − 2	24 – –	30 – –	27 22 5	21 17 4	33 28 5	5.6
**Apprehension**	T0 T1	N –	N N	P –	P –	P P	N N	N N	X
**Relocation**	T0 T1	N –	N N	P –	P –	P P	N N	N N	X
**SJPS** (°) *Change score*	T0 T1	5.19 – –	3.64 3.19 0.45	1.69 – –	1.15 – –	2.61 0.60 2.01	3.41 2.21 1.20	**6.58** **2.20** **4.38**	3
**COPL 1**^**a**^ Both arms (mm) *Change score*	T0 T1	320 – –	218 150 68	280 – –	291 – –	246 157 89	164 177 − 13	275 258 17	95.8
**COPL 2**^**b**^ Non-injured arm (mm) *Change score*	T0 T1	462 – –	291 201 90	371 – –	283 – –	**322** **189** **133**	193 200 − 7	344 306 38	108.9
**COPL 3**^**c**^ Injured arm (mm) *Change score*	T0 T1	445 – –	**316** **178** **138**	394 – –	299 – –	**331** **201** **130**	189 174 15	296 304 − 8	78.4
**CS-iMVC** Non-injured arm (kg) *Change score*	T0 T1	7.0 – –	15.7 17.2 -1.5	8.4 – –	16.2 – –	13.2 13.6 0.4	10.7 10.6 0.1	7.1 5.6 1.5	X
**CS-iMVC** Injured arm (kg) *Change score*	T0 T1	8.6 – –	14.7 15.2 -0.5	8.1 – –	13.0 – –	10.9 12.3 − 1.4	7.4 8.2 − 0.8	8.1 7.4 0.7	X
**Patient satisfaction** **(1–5)**	T1	–	1	1	1	1	1	1	X
**Perceived change (1–7)**	T1	–	3	**2**	3	3	**1**	**2**	2

*MDC* minimal detectable change, *WOSI* Western Ontario Shoulder Instability Index, *TSK* Tampa Scale of Kinesiophobia, *SJPS* shoulder joint position sense, *COPL* center of pressure path length, *CS-iMVC* Constant Score-Isometric Maximal Voluntary Contraction, *N* negative, *P* positive

Results in bold indicate change higher than MDC. * = drop-out. – = Post-test data not collected. X = MDC unknown or not relevant. Patient satisfaction: 1 = satisfied, 5 = Dissatisfied. Perceived change: 1 = Fully recovered, 4 = Unchanged, 7 = Much worse

^a^Both arms, eyes closed

^b^Non-injured shoulder, eyes open

^c^Injured shoulder, eyes open

## Discussion

Data was collected through observations and interviews on five areas of feasibility: recruitment, retention, compliance, acceptability and safety. Recruitment processes were slow and only seven of the desired number of 20 participants were recruited. Two participants dropped out of the study, and one could only complete 8 weeks of the intervention. Two of seven participants achieved predefined acceptable compliance. All participants expressed satisfaction with the intervention and felt safe during the exercises. Four adverse events occurred, one of which was related to the intervention (patellar redislocation). All participants improved in the GPE. Change greater than minimal detectable change (MDC) was reported in four participants in some of the outcome assessments. One of the seven participants declined surgery.

### Recruitment

The desired number of participants for the present study was 20, but only 7 were recruited. Unfortunately, the reasons why only 7 of 15 patients volunteered for the study is not properly recorded. Exact data regarding how many patients were screened for eligibility, how many were considered eligible and why some eligible patients chose not to participate in the project was not collected. This would have been useful information when considering how to increase recruitment, and it is advised that data on these areas is collected before the following RCT. The surgeons responsible for recruiting had the impression that lack of willingness to participate in the study might be that the patients already had been through a training program, or that they simply could not spare the time for a 3-month intervention, though this was not confirmed. In order to increase the number of participants, the recruitment period should have been extended to approximately 15 months, which was not possible due to practical limitations. In a future RCT, the slow recruitment process should be taken into account to ensure the desired number of participants is reached. Other ways of optimizing recruitment include an increase in recruitment pathways and locations (e.g. through more hospitals/clinics), but also a more ongoing recruitment strategy would probably have been helpful.

### Retention

One participant only completed 8 weeks of the intervention, due to a pre-planned surgery. For a future study, it is recommended that participants who are not able to complete all 12 weeks of the intervention are excluded. However, due to the low number of participants in the present study, 8 weeks of participation was deemed sufficient to gather feasibility data and the participant was therefore included. Two participants dropped out during the study, one because of a fall and the other for unknown reasons. As these causes for drop-out are hard to foresee and prevent, it is recommended that a future study takes possible drop-out into account when establishing the desired number of participants. A trustful and friendly atmosphere is essential for retention [39]. Based on patient feedback, this was achieved in the present study. It therefore seems unlikely that this was the cause of the two drop-outs.

### Compliance

Possible barriers for successful compliance, especially regarding home exercises, is time and equipment needed to perform the exercises [40, 41]. Two of seven participants achieved predefined acceptable compliance in this study. Barriers for successful compliance were inability to take along exercise equipment when travelling, sick leave, holidays and lack of time/busy days. Home exercise options without equipment might mitigate this barrier, but completing the exercises will still be time-consuming. To increase compliance, adjustments to the SINEX program may be beneficial. However, this might impact the effectiveness of the program, and should be further researched. Other barriers to successful compliance may be travel distance and expenses to attend supervised physiotherapy sessions [40, 41], but this did not impact our study, since all the participants achieved the minimum recommended compliance for supervised sessions.

### Acceptability

Based on data gathered in the interviews and the GPE, all participants expressed satisfaction with the intervention and experienced improved shoulder function, despite few detectable improvements in the measurement outcomes. Questionnaires about global perceived effect are rarely validated [29], and it remains uncertain whether the change expressed by participants in this questionnaire is representative for intervention efficacy. Still, many clinicians would not consider an intervention to be effective unless the patients themselves perceived a change [29].

### Safety

Four adverse events occurred, one of which was related to the intervention (patellar redislocation). All participants felt safe during the exercises and the whole intervention. Two participants experienced a redislocation of the shoulder during the intervention period, but since these events occurred outside the project, they cannot be attributed to the current intervention. However, to avoid such events in the future, vigorous activities ought to be restricted during the intervention period. One participant experienced patellar a redislocation during the exercises. Due to hypermobile joints, the participant experienced dislocations in everyday life. Patients with generalized hypermobility may therefore need additional attention and adjustments to reduce the risk of adverse events.

Although all participants expressed that they felt safe during the intervention, they called for exercises that would improve their feeling of safety in everyday life.

Based on the injury mechanism of shoulder dislocations, age and symptoms, traumatic shoulder dislocation may be compared with anterior cruciate ligament (ACL) tear in the knee, and rehabilitation principles may be based on the same neuromuscular exercise principles [32]. Rehabilitation of patients with ACL injuries includes the simulation of real-case sport activity scenarios via proactive and reactive exercises [42], a component SINEX only incorporated to some extent (few exercises, and only within limited ROM with low resistance). Since the participants in our study expressed concerns about unexpected movements, such exercises may reduce the fear of redislocations, and potentially their actual incidence. It therefore seems feasible to supplement SINEX with exercises simulating real-case sport scenarios, including unexpected movements with safe falling or learning of falling techniques, and gradually increase ROM and resistance, especially for patients returning to high-performance sports.

### Outcome assessments

Many of our participants had low WOSI and TSK baseline scores (meaning good function), indicating that there was little room for improvement. This may explain the low WOSI and TSK change scores in our study. However, as WOSI originally was designed to capture treatment effects in shoulder instability patients before and after surgery, it was surprising that the current sample displayed such good patient-reported shoulder function at pre-testing, simultaneously with eligibility for surgery. One explanation, as suggested by the participants themselves, could be that WOSI only asks about symptoms during the past week. As many of the participants had given up certain sports or activities, they had not experienced instability symptoms in the week before and therefore scored low on several items. But since the score did not reflect the fact that they had given up sports or other activities, their low scores may not reflect their real activity problem/have been somewhat incorrect. This suggests that WOSI may not be sensitive to *all* shoulder instability patients, and supplementary assessment regarding the questionnaire’s time frame may be needed before including WOSI in an RCT.

Since the SINEX program aimed at increasing neuromuscular control, several measurements of shoulder proprioception and sensorimotor control were included as objective outcome methods (SJPS, force platform in prone lying), all thoroughly rehearsed before study start, and performed by only two persons. Only one participant showed improvement above MDC on SJPS, a result that must be interpreted judiciously since the reliability of this test at 90° of shoulder flexion has been reported to be poor [33]. As previously described, the selected SJPS test was performed near 90° because the SINEX program included proprioceptive exercises mostly near/above 90°. In addition, movements within this range are considered more representative of daily activities. Two of the participants improved above MDC on the force platform, indicating future promising results for this method. However, since MDC for this test has only been researched in a healthy population, the results for shoulder instability patients must be interpreted with caution. None of the participants improved in CS-iMVC strength. As previously described, MDC for this test has only been established for the entire Constant Score protocol [35]. The degree of improvement in isolated CS-iMVC strength required to be considered a real change is not known. Since efficacy testing was not a primary goal of this study, results must be interpreted with caution due to the small sample size. However, since MDC for several of the outcome assessments is unknown, it is advised that these are established and suitability of the outcome assessments further explored before moving on to an RCT.

### Study limitations and strengths

One of the limitations of our study is that conclusions on the effectiveness of the SINEX program are not possible to draw, due to the lack of a control group receiving standard physical therapy or surgery. This, however, was a deliberate decision, since the study design primarily intended to evaluate feasibility rather than effectiveness.

Another limitation of the study is the small sample size, as the desired number of participants was not reached due to practical limitations. This implies that the outcomes of this study must be interpreted with caution. However, the combination of quantitative and qualitative data ensures a deeper understanding of the participants’ experience with the study and the intervention. This argues that data regarding the feasibility of the study should be taken into account when moving forward towards an RCT. Data regarding why some patients chose not to partake in the project was not collected. This is unfortunate, as such data might have offered suggestions as how to improve recruitment in a future RCT. A stakeholder analysis interviewing patients who chose not to participate in the study along with the recruiting surgeons would be helpful before moving forward with an RCT. Since it is possible that the burden of participation is too high to be feasible on a larger scale speaking to the recruited patients would help to elucidate this.

The strength of our study lies in its adherence to strict criteria for feasibility and safety studies [22], the use of a standardized exercise program and protocol setup for shoulder instability patients, and the use of both qualitative and quantitative outcome assessments, including both patient-reported and thoroughly rehearsed objective measurements based on a standardized protocol.

## Conclusions

The present study suggests that further assessment is required on several areas before performing efficacy testing of the SINEX for patients with TASI considered eligible for surgery in a RCT. No adverse events suggest that the program is safe regarding the affected shoulder, but participants with general hypermobility may need additional adjustments to prevent adverse events in other areas of the body. It is recommended that sports activities during the intervention may be restricted to minimize the risk of redislocation, which may also affect retention of participants. Before a future RCT, we suggest that the SINEX program is optimized, that MDC for all outcome measures is established, and that recruitment strategies are improved. During an RCT we recommend that recruitment is more thoroughly followed up to increase likelihood of reaching desired sample size.

### Implications for physiotherapy practice

The SINEX program for patients with TASI not considered eligible for surgery is designed to be used as is, without further training of the physiotherapists. Our experience in administering this intervention was that it is feasible to use based on the written instructions. Should it also prove effective in patients with TASI considered eligible for surgery, the exercise program would be beneficial for physiotherapists treating this patient group.

## Supplementary information

**Additional file 1.** The SINEX-Protocol in Norwegian.

## Data Availability

All data generated or analysed during this study are included in this published article (and its supplementary information files).

## Abbreviations

TASI: Traumatic anterior shoulder instability
SINEX: Shoulder Instability Neuromuscular Exercise
RCT: Randomized controlled trial
WOSI: Western Ontario Shoulder Instability Index
TSK: Tampa Scale of Kinesiophobia
RM: Repetition maximum
MDC: Minimal detectable change
SJPS: Shoulder joint position sense
COPL: Center of pressure path length
CS-iMVC: Constant Score-Isometric Muscular Voluntary Contraction
ROM: Range of motion
ACL: Anterior cruciate ligament
GPE: Global perceived effect questionnaire

## References

[1] Liavaag S, Svenningsen S, Reikeras O (2011). The epidemiology of shoulder dislocations in Oslo. Scand J Med Sci Sports..

[2] Sofu H, Gursu S, Kockara N, Oner A, Issin A, Camurcu Y (2014). Recurrent anterior shoulder instability: review of the literature and current concepts. World J Clin Cases..

[3] Pope EJ, Ward JP, Rokito AS (2011). Anterior shoulder instability—a history of arthroscopic treatment. Bull NYU Hosp Jt Dis..

[4] Jaggi A, Lambert S (2010). Rehabilitation for shoulder instability. Br J Sports Med..

[5] Godin J, Sekiya JK (2010). Systematic review of rehabilitation versus operative stabilization for the treatment of first-time anterior shoulder dislocations. Sports Health..

[6] Blomquist J. Surgical treatment of shoulder instability in Norway: the Norwegian Shoulder Instability Register [dissertation]. Bergen: University of Bergen; 2016. p. 108. .

[7] Monk AP, Garfjeld Roberts P, Logishetty K (2015). Evidence in managing traumatic anterior shoulder instability: a scoping review. Br J Sports Med..

[8] Eljabu W, Klinger H, von Knoch M (2017). The natural course of shoulder instability and treatment trends: a systematic review. J Orthopaed Traumatol..

[9] Handoll HH, Almaiyah MA, Rangan A (2004). Surgical versus non-surgical treatment for acute anterior shoulder dislocation. Cochrane Database Syst Rev..

[10] Longo UG, Loppini M, Rizzello G, Ciuffreda M, Maffulli N, Denaro V (2014). Management of primary acute anterior shoulder dislocation: systematic review and quantitative synthesis of the literature. Arthroscopy..

[11] Ma R, Brimmo OA, Li X, Colbert L (2017). Current concepts in rehabilitation for traumatic anterior shoulder instability. Curr Rev Musculoskelet Med..

[12] Rugg CM, Hettrich CM, Ortiz S, Wolf BR, Zhang AL, Group MSI (2018). Surgical stabilization for first-time shoulder dislocators: a multicenter analysis. J Shoulder Elbow Surg..

[13] Bottoni CR, Wilckens JH, DeBerardino TM (2002). A prospective, randomized evaluation of arthroscopic stabilization versus nonoperative treatment in patients with acute, traumatic, first-time shoulder dislocations. Am J Sports Med..

[14] Brophy RH, Marx RG (2009). The treatment of traumatic anterior instability of the shoulder: nonoperative and surgical treatment. Arthroscopy..

[15] Ageberg E, Nilsdotter A, Kosek E, Roos EM (2013). Effects of neuromuscular training (NEMEX-TJR) on patient-reported outcomes and physical function in severe primary hip or knee osteoarthritis: a controlled before-and-after study. BMC Musculoskeletal Disorders..

[16] Eitzen I, Moksnes H, Snyder-Mackler L, Risberg MA (2010). A progressive 5-week exercise therapy program leads to significant improvement in knee function early after anterior cruciate ligament injury. J Orthop Sports Phys Ther..

[17] Stensrud S, Risberg MA, Roos EM (2015). Effect of exercise therapy compared with arthroscopic surgery on knee muscle strength and functional performance in middle-aged patients with degenerative meniscus tears. Am J Phys Med Rehabil..

[18] Warby SA, Ford JJ, Hahne AJ (2018). Comparison of 2 exercise rehabilitation programs for multidirectional instability of the glenohumeral joint: a randomized controlled trial. Am J Sports Med..

[19] Watson L, Balster S, Lenssen R, Hoy G, Pizzari T (2018). The effects of a conservative rehabilitation program for multidirectional instability of the shoulder. J Shoulder Elbow Surg..

[20] Eshoj H, Rasmussen S, Frich LH (2017). A neuromuscular exercise programme versus standard care for patients with traumatic anterior shoulder instability: study protocol for a randomised controlled trial (the SINEX study). Trials..

[21] Eldridge SM, Chan CL, Campbell MJ (2016). CONSORT 2010 statement: extension to randomised pilot and feasibility trials. Pilot Feasibility Stud..

[22] Polit DF, Beck CT (2017). Feasibility assessments and pilot tests of interventions using mixed methods. Nursing Research, Generating and Assesing Evidence for Nursing Practice. 10 ed.

[23] Kirkley A, Griffin S, McLintock H, Ng L (1998). The development and evaluation of a disease-specific quality of life measurement tool for shoulder instability. The Western Ontario Shoulder Instability Index (WOSI). Am J Sports Med..

[24] Skare O, Liavaag S, Reikeras O, Mowinckel P, Brox JI (2013). Evaluation of Oxford instability shoulder score, Western Ontario shoulder instability index and Euroqol in patients with SLAP (superior labral anterior posterior) lesions or recurrent anterior dislocations of the shoulder. BMC Res Notes..

[25] Mintken PE, Cleland JA, Whitman JM, George SZ (2010). Psychometric properties of the Fear-Avoidance Beliefs Questionnaire and Tampa Scale of Kinesiophobia in patients with shoulder pain. Arch Phys Med Rehabil..

[26] Vlaeyen JW, Kole-Snijders AM, Boeren RG, van Eek H (1995). Fear of movement/(re) injury in chronic low back pain and its relation to behavioral performance. Pain..

[27] Hapidou EG, O'Brien MA, Pierrynowski MR, de Las HE, Patel M, Patla T (2012). Fear and avoidance of movement in people with chronic pain: psychometric properties of the 11-Item Tampa Scale for Kinesiophobia (TSK-11). Physiother Can..

[28] Haugen AJ, Grovle L, Keller A, Grotle M (2008). Cross-cultural adaptation and validation of the Norwegian version of the Tampa scale for kinesiophobia. Spine (Phila Pa 1976)..

[29] Ostelo RW, de Vet HC (2005). Clinically important outcomes in low back pain. Best Pract Res Clin Rheumatol..

[30] Hegedus EJ, Goode AP, Cook CE (2012). Which physical examination tests provide clinicians with the most value when examining the shoulder? Update of a systematic review with meta-analysis of individual tests. Br J Sports Med..

[31] Lo IK, Nonweiler B, Woolfrey M, Litchfield R, Kirkley A (2004). An evaluation of the apprehension, relocation, and surprise tests for anterior shoulder instability. Am J Sports Med..

[32] Eshoj H (2016). Non-operative Treatment, Outcome Measurements and Characteristics of Patients with Traumatic Anterior Shoulder Dislocation.

[33] Vafadar AK, Cote JN, Archambault PS (2016). Interrater and intrarater reliability and validity of 3 measurement methods for shoulder-position sense. J Sport Rehabil..

[34] Edouard P, Gasq D, Calmels P, Degache F (2014). Sensorimotor control deficiency in recurrent anterior shoulder instability assessed with a stabilometric force platform. J Shoulder Elbow Surg..

[35] Moeller AD, Thorsen RR, Torabi TP, et al. The Danish version of the modified Constant-Murley shoulder score: reliability, agreement, and construct validity. J Orthop Sports Phys Ther. 2014;5(44):336–40. 10.2519/jospt.2014.5008 24673447

[36] Raastad T, Ronnestad BR (2010). Adaptasjon til styrketrening. Styrketrening - i teori og praksis.

[37] Pasienttilfredshet. 2016; https://oslo-universitetssykehus.no/generiske-skjemaer%2D%2Dpasienttilfredshet. Accessed 29 Sept 2020.

[38] Malterud K (2012). Systematic text condensation: a strategy for qualitative analysis. Scand J Public Health..

[39] Blanton S, Morris DM, Prettyman MG (2006). Lessons learned in participant recruitment and retention: the EXCITE trial. Phys Ther..

[40] Ross S, Grant A, Counsell C, Gillespie W, Russell I, Prescott R (1999). Barriers to participation in randomised controlled trials: a systematic review. J Clin Epidemiol..

[41] Walsh E, Sheridan A (2016). Factors affecting patient participation in clinical trials in Ireland: a narrative review. Contemp Clin Trials Commun..

[42] Davies GJ, McCarty E, Provencher M, Manske RC (2017). ACL Return to Sport Guidelines and Criteria. Curr Rev Musculoskelet Med..

