# Supplementary material for: Feasibility of the SINEX program for patients with traumatic anterior shoulder instability
Source: Pilot Feasibility Stud. 2020 Oct 6;6:148. doi: 10.1186/s40814-020-00679-x (PMC7541274; doi:10.1186/s40814-020-00679-x)
Supplement: Supplementary file 1 — Additional file 1. The SINEX-Protocol in Norwegian. [file 40814_2020_679_MOESM1_ESM.pdf]

Øvelse 1 – Kontroll og «setting» av skulderbladet				
**Biomekanisk hensikt:** Å optimalisere posisjonering og bevegelse av skulderbladet.				
**Praktisk hensikt:** Tilstrekkelig kontroll over skulderbladet er avgjørende for god skulderfunksjon.				
**Generelt:** Unngå å heise skuldrene opp til ørene og overaktivering av muskuler i nakke og øvre skulder.				
**Grunnleggende nivå**				
**Nivå**	**Utførelse**	**Bilde**	**Dosering**	**Kommentarer**
1A	Ligg på magen med armene langs siden.<br><br>Aktiver musklene nedenfor og mellom skulderbladene for å «sette» skulderbladet.<br><br>Tell til fem, slipp opp, og gjenta.	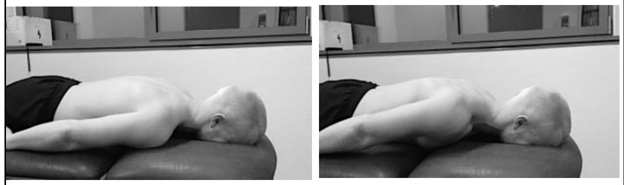	20-25 reps<br>2 sett<br>Daglig	Pass på at skuldrene ikke «heises opp» mot ørene underveis i øvelsen.
1B	Sitt oppreist på en fitnessball med begge beina i bakken. Nakke og rygg i nøytralstilling. Armene hviler på lårene.<br><br>Aktiver musklene nedenfor og mellom skulderbladet for å «sette» skulderbladet.<br><br>Behold «settingen» av skulderbladet mens du roterer hodet sakte fra side til side.	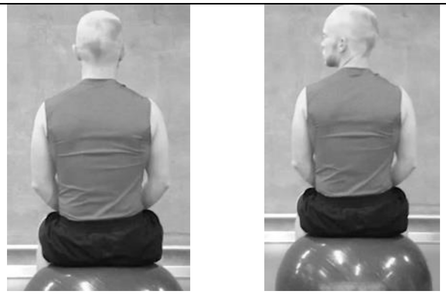	20-25 reps<br>2 sett<br>Daglig	Pass på at skuldrene ikke «heises opp» mot ørene underveis i øvelsen.
1C	Startposisjon som i B. «Setting» av skulderbladet som i B.<br><br>Behold «settingen» av skulderbladet mens du sakte strekker armene opp mot taket. Ha tomlende vendt oppover. Armene skal forme en bred V.<br><br>Senk armene ned igjen, slapp av, og gjenta.	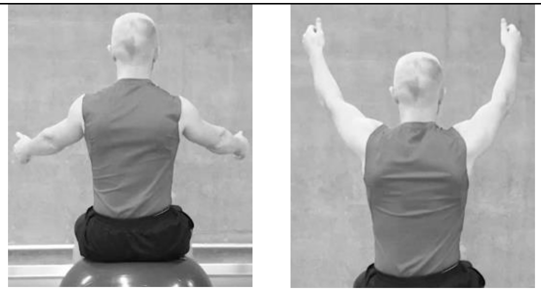	20-25 reps<br>2 sett<br>Daglig	Om nødvendig kan du bruke et speil for å se at øvelsen utføres korrekt.

1D	Startposisjon som i C. Løft benet på samme side som instabil skulder. Utfør så øvelsen som i C.	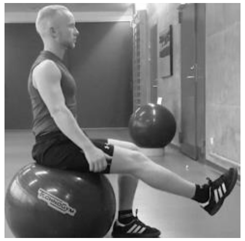 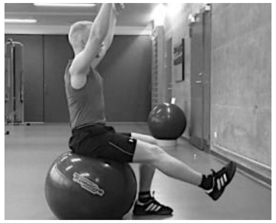	20-25 reps<br>2 sett<br>Daglig	Om nødvendig kan du bruke et speil for å se at øvelsen utføres korrekt.
1E	Startposisjon som i C. Plasser den ene enden av strikken under foten som er motsatt av den instabile skulderen.<br><br>Behold «settingen» av skulderbladet mens du løfter armene mot taket som i C. Den instabile armen holder i strikken.<br><br>Senk armene mens du har kontroll over skulderbladet, slapp av, og gjenta.	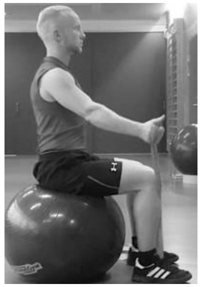 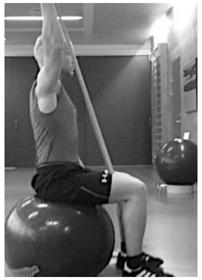	20-25 reps<br>2 sett<br>Daglig	Om nødvendig kan du bruke et speil for å se at øvelsen utføres korrekt.

Elite nivå				
1F	Stå på begge beina med ryggen i nøytral stilling. Stå på den ene enden av strikken med foten motsatt av instabil skulder.<br><br>«Sett» skulderbladet. Hold i strikken med instabil side og strekk armen mot taket rett foran deg. Senk armen deretter litt ned igjen, stram strikken ved å skyve frem med motsatt arm (bilde 2), og løft armen opp mot taket igjen. Slipp så opp i strikken, senk armen helt ned, slapp av og gjenta.	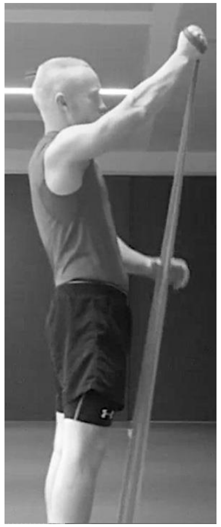 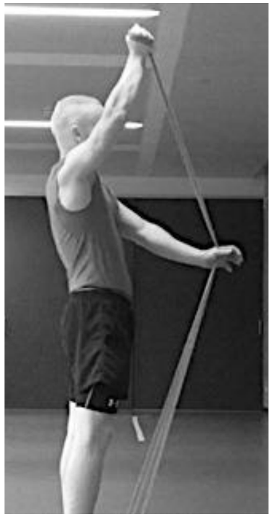	8-12 reps<br>2 sett	Om nødvendig kan du bruke et speil for å se at øvelsen utføres korrekt.
1G	Som F, men med beinet på instabil side løftet fra gulvet.	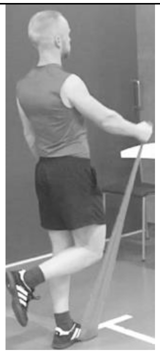 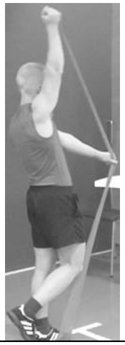	8-12 reps<br>2 sett	Om nødvendig kan du bruke et speil for å se at øvelsen utføres korrekt.

Øvelse 2 – Kontroll og «setting» av skulderkulen (innadrotasjon)

Biomekanisk hensikt: Å optimalisere posisjonering og bevegelse av skulderleddet ved rotasjonsbevegelser.

Praktisk hensikt: Redusert kontroll og uhensiktsmessig posisjonering av skulderkulen i skulderleddet kan ofte resultere i smerter og ubehag. Denne øvelsen er derfor viktig for å sørge for god posisjonering og bevegelse av skulderleddet.

Generelt: Unngå å trekke skulderbladene for mye sammen bak på ryggen ved å overaktivere i musklene mellom skulderbladene.

Grunnleggende nivå

Nivå	Utførelse	Bilde	Dosering	Kommentarer
2A	Ligg på rygg med beina bøyd. Plasser armen 45 grader ut fra kroppen med 90 graders bøy i albuen. Legg et sammenbrettet håndkle under albuen slik at overarmen blir horisontal.<br><br>Øvelsen utføres ved at du trekker skulderkulen litt inn i skulderleddet. Tell til fem, slapp av, og gjenta.	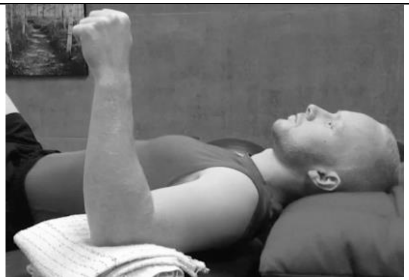	20-25 reps<br>2 sett<br>Daglig	Øvelsen utføres korrekt når skulderen bevegeser nedover mot underlaget. Du kan kjenne etter med fingrene på motsatt hånd ved å plassere dem på skulderen.<br><br>Det skal være en liten bevegelse, så det er ikke meningen at skulderbladet skal bevege seg opp eller ned.
2B	Startposisjon som i A. «sett» skulderleddet som i A.<br><br>Roter overarmen frem og tilbake mens du holder aktiveringen som i A.	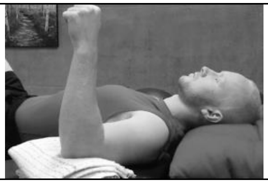 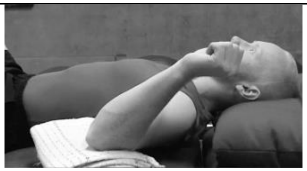	20-25 reps<br>2 sett<br>Daglig	Som over.
2C	Ligg på rygg med beina bøyd. Armen plasseres 90 grader ut fra kroppen med 90 grader bøy i albuen. Plasser et sammenbrettet håndkle under albuen slik at overarmen blir horisontal.<br><br>«Sett» skulderleddet. Roter armen innover med motstand fra en strikk som du fester ved bakkenivå på linje med hånden din. Returner tilbake til utgangsposisjon med kontroll, og gjenta.	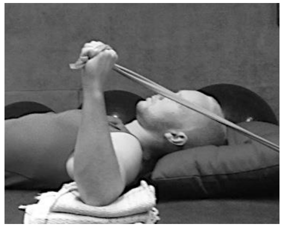 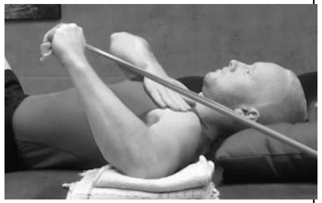	20-25 reps<br>2 sett<br>Daglig	Som over.

2D	Startposisjon som i C, men uten håndkle under albuen. Albuen skal holdes oppe slik at den svever i luften.<br><br>«Sett» skulderleddet. Roter overarmen innover og tilbake til nøytral med kontroll, og gjenta.	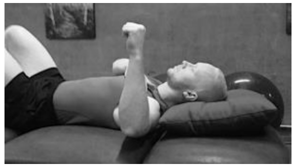 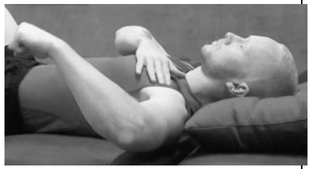	20-25 reps<br>2 sett<br>Daglig	Som over.
2E	Fest den ene enden av strikken bak kroppen litt over skulderhøyde. Hold i strikken med den instabile armen.<br><br>Stå med motsatt fot av instabil arm fremst, knærne lett bøyd, kroppsvekten forskjøvet til fremste ben og kneet i linje med hoften og foten.<br><br>Plasser overarmen 90 grader ut fra kroppen med 90 grader bøy i albuen med hånden pekende mot taket. «Sett» skulderleddet.<br><br>Roter overarmen innover (hånden peker fremover). Returner til nøytral med kontroll, og gjenta.	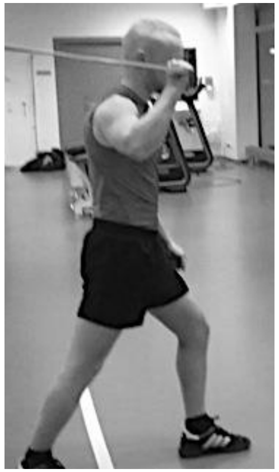 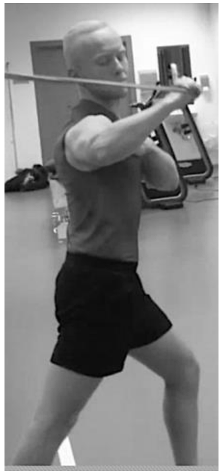	20-25 reps<br>2 sett<br>Daglig	

Elite nivå				
2F	Som i E, men med armen 120-130 grader ut fra kroppen (kasteposisjon).	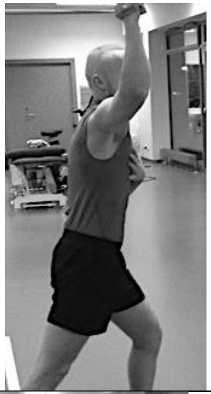 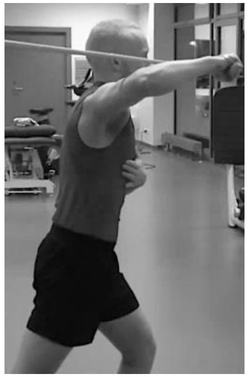	8-12 reps<br>2 sett<br>Tre ganger i uken	
2G	Som i F, men en fot i luften. Stå på det motsatte benet av instabil skulder.<br><br>Dersom du trenger enda større utfordring: Løft armen ytterligere, øk hastigheten og motstanden, og/eller lukk øynene. Introduser en progresjon av gangen.	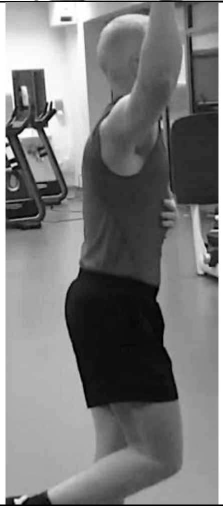 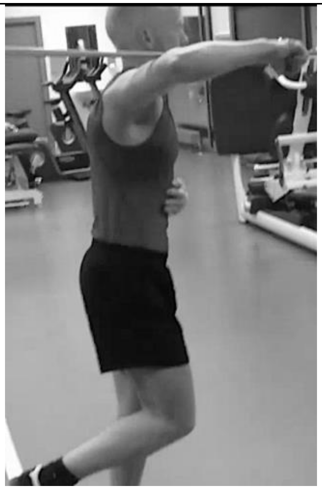	8-12 reps<br>2 sett<br>Tre ganger i uken	

Øvelse 3 – Kontroll og «setting» av skulderkulen (utadrotasjon)

Biomekanisk hensikt: Å optimalisere posisjonering og bevegelse av skulderleddet ved rotasjonsbevegelser.

Praktisk hensikt: Redusert kontroll og uhensiktsmessig posisjonering av skulderkulen i skulderleddet kan ofte resultere i smerter og ubehag. Denne øvelsen er derfor viktig for å sørge for god posisjonering og bevegelse av skulderleddet.

Generelt: Unngå å trekke skulderbladene for mye sammen bak på ryggen ved å overaktivere i musklene mellom skulderbladene.

Grunnleggende nivå

Nivå	Utførelse	Bilde	Dosering	Kommentarer
3A	Denne øvelsen er den samme som 2A, og du trenger bare gjøre den én gang.	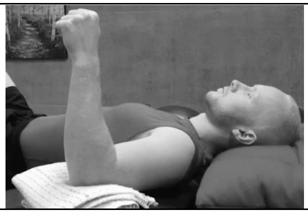	20-25 reps<br>2 sett<br>Daglig	Som i 2A
3B	Denne øvelsen er den samme som 2B, og du trenger bare gjøre den én gang.	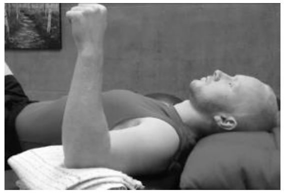 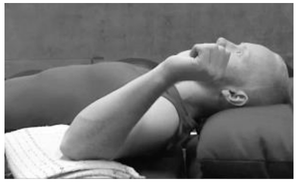	20-25 reps<br>2 sett<br>Daglig	Som i 2B
3C	Ligg på rygg med beina bøyd. Armen plasseres 90 grader ut fra kroppen med 90 grader bøy i albuen. Plasser et sammenbrettet håndkle under albuen slik at overarmen blir horisontal.<br><br>«Sett» skulderleddet. Roter armen utover med motstand fra en strikk som du fester ved bakkenivå på linje med hånden din. Returner tilbake til utgangsposisjon med kontroll, og gjenta.	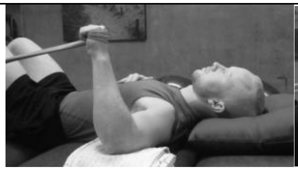 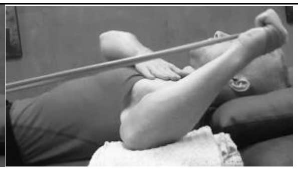	20-25 reps<br>2 sett<br>Daglig	Som 2C

3D	Denne øvelsen er den samme som 2D, og du trenger bare gjøre den én gang.	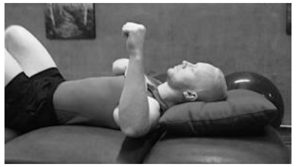 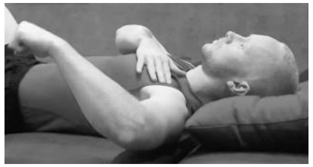	20-25 reps<br>2 sett<br>Daglig	Som i 2D.
3E	Fest den ene enden av strikken foran kroppen i hoftehøyde. Hold den andre enden av strikken med instabil arm. Startposisjon som i 2E, men med armen pekende fremover i stedet for oppover.<br><br>«Sett» skulderleddet. Roter overarmen bakover til hånden peker mot taket. Behold kontrollen over skulderen mens du roterer tilbake. Gjenta.	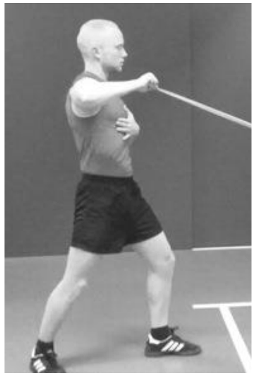 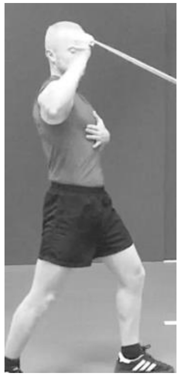	20-25 reps<br>2 sett<br>Daglig	Som i 2E.

**Elite nivå**				
3F	Som i 3E, men med armen 120-130 grader ut fra kroppen (kasteposisjon).	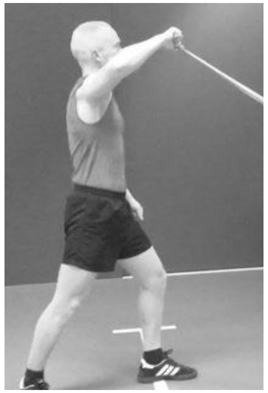 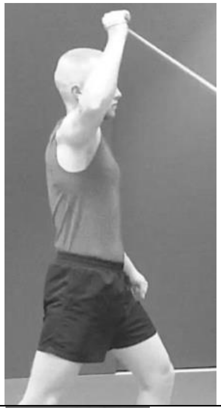	8-12 reps<br>2 sett<br>Tre ganger i uken	Som i 2F
3G	Som i 3F, men ett ben i luften. Stå på det motsatte benet av den instabile skulderen.<br><br>Dersom du trenger enda større utfordring: Løft armen ytterligere, øk hastigheten og motstanden, og/eller lukk øynene. Introduser en progresjon av gangen.	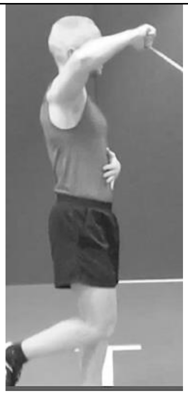 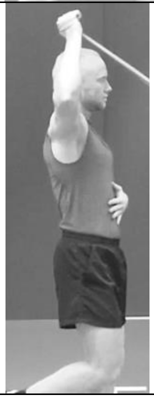	8-12 reps<br>2 sett<br>Tre ganger i uken	Som i 2G

**Øvelse 4 – Koaktivering av skuldermuskler**				
**Biomekanisk hensikt:** Å optimalisere samtidig aktivering av skuldermuskler, kjernemuskulatur og leddsans.				
**Praktisk hensikt:** I skulderen er det mange muskler som jobber sammen for at skulderen skal være stabil og jobbe effektivt i ulike aktiviteter gjennom hverdagen. Disse øvelsene har til hensikt å optimalisere samarbeidet mellom disse musklene, samt kjernemusklene dine.				
**Generelt:** Sørg for å aktivere magemuskulatur for å holde en stabil og nøytral rygg gjennom alle øvelsene.				
**Grunnleggende nivå**				
**Nivå**	**Utførelse**	**Bilde**	**Dosering**	**Kommentarer**
4A	Ligg i en planke-stilling med en fitnessball som støtte under lårene.<br><br>Skyv armene ned mot gulvet, slik at skulderbladene sklir fremover. Dette kalles en push-up plus. Hold stillingen mens du ruller bakover, slapp av, kom frem og gjenta.	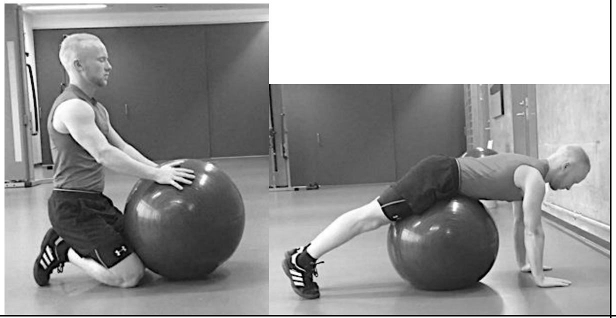	20-25 reps<br>2 sett<br>Daglig	
4B	Startposisjon som i A. Gjør en push-up plus.<br><br>Hold stillingen mens du forskyver vekten fra side til side.<br><br>Unngå å «henge» på skuldrene.	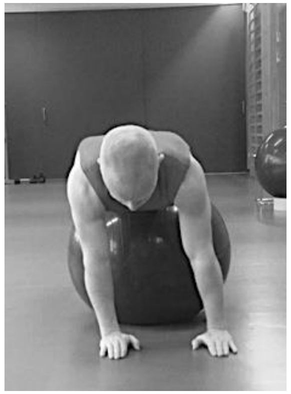 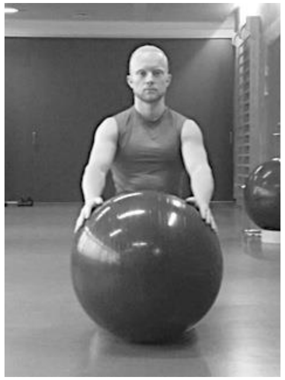	20-25 reps<br>2 sett<br>Daglig	

4C	Stå med beina i hoftebreddes avstand. Len deg fremover mot en fitnessball plassert i brysthøyde i et hjørne.<br><br>Aktiver musklene rundt skulderbladet og gjøre en push-up plus. Hold stillingen mens du løfter en og en arm.	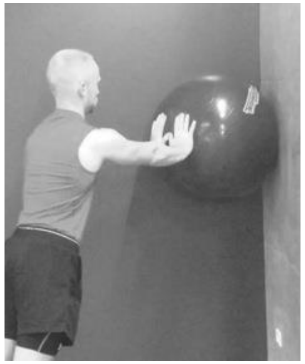 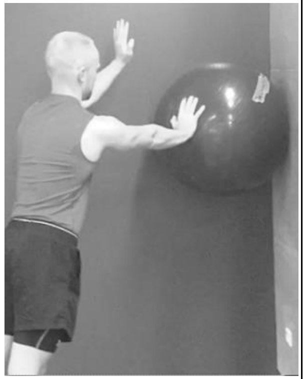	20-25 reps<br>2 sett<br>Daglig	
4D	Ligg i en planke-stilling med en fitnessball som støtte under lårene.<br><br>Gjør en push-up plus. Hold stillingen mens du går fremover med begge armene.<br><br>Rull tilbake og gjenta.	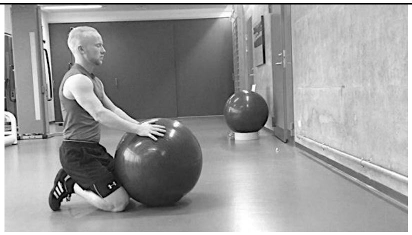 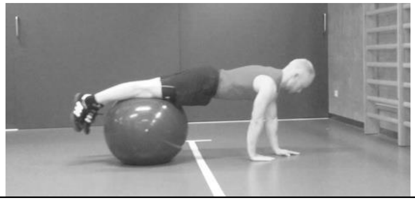	20-25 reps<br>2 sett<br>Daglig	

4E	Startposisjon som i D.<br><br>Gå fremover med begge armene.<br><br>Bøy i albuene og gjør en armheving. Gjør en push-up plus på toppen av armhevingen.<br><br>Rull tilbake og gjenta.	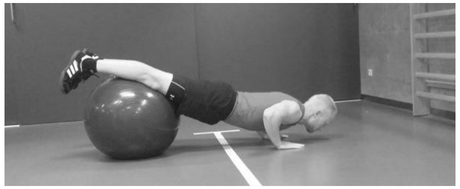<br>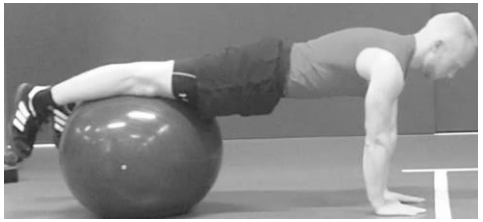	20-25 reps<br>2 sett<br>Daglig	
**Elite nivå**				
4F	Startposisjon som i E.<br><br>Gjør en armheving som i E, men med så stor fart opp at du får et hopp på toppen.<br><br>Sørg for at du har kontroll over skulderbladet i landingen.<br><br>Gjenta.	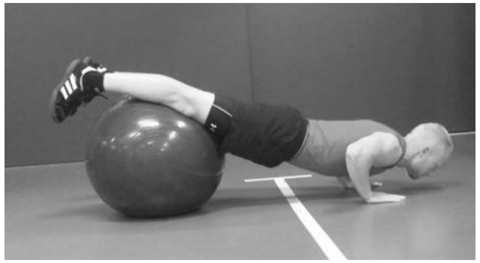<br>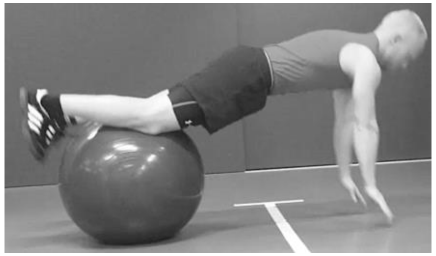	8-12 reps<br>2 sett<br>Tre ganger i uken	

4G	Stå i en planke-stilling.<br><br>Gjør en armheving, og skyv opp med så stor fart at både hender og føtter letter fra bakken.<br><br>Sørg for at du har kontroll over skulderbladet i landingen.<br><br>Gjenta.<br><br>Ved behov for videre progresjon:Hold i en strikk som er festet i skulderhøyde til siden for kroppen. Hold strikken med instabil arm.	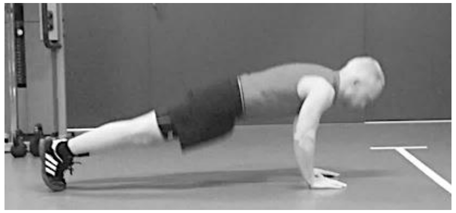<br>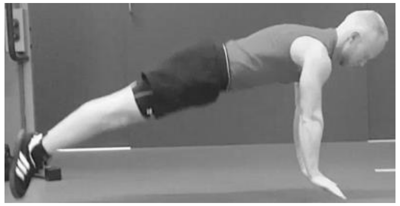	8-12 reps<br>2 sett<br>Tre ganger i uken	

Øvelse 5 – Dynamisk muskulær stabilitet i skulderleddet

Biomekanisk hensikt: Å optimalisere den dynamiske stabiliteten i skulderen med samtidig kontroll over skulderbladet og skulderleddet.

Praktisk hensikt: Denne øvelsen er viktig for å forbedre din evne og dine ferdigheter ved raske bevegelser i skulderen, noe som ofte forekommer i hverdagen, og særlig i sport.

Generelt:

Grunnleggende nivå

Nivå	Utførelse	Bilde	Dosering	Kommentarer
5A	Sitt oppreist på en fitnessball med begge føttene i bakken. Nakken og ryggen er i nøytral stilling. Legg et sammenbrettet håndkle melom albuen og kroppen.<br><br>«Sett» skulderbladet og skluderleddet.<br><br>Bruk motsatt arm til å lage motstand, og dra utover med instabil arm. Det skal kun skje en aktivering, ikke en bevegelse. Hold 10 sekunder, slapp av og gjenta.	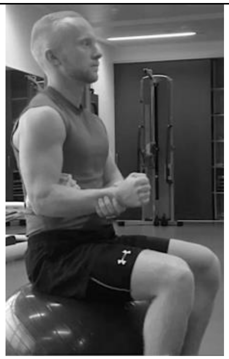	10 sek<br>3 sett<br>Daglig	Unngå å heise skulderen opp til øret.
5B	Fest enden på en strikk i albuehøyde på motsatt side av instabil skulder, og hold den andre enden i hånden med instabil arm. Startposisjon som i A.<br><br>Med stram strikk, gjennomfør små rytmiske bevegelser fra side til side mens du holder skulderbladet og skulderen stabile. Slapp av og gjenta.	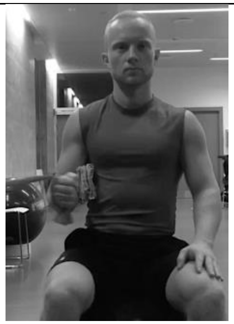	10 sek<br>3 sett<br>Daglig	Merk: Bildet viser strikken festet på feil side. Strikken skal festes på motsatt side av instabil skulder.<br><br>Unngå å heise skulderen opp til øret.

5C	Startposisjon som i B. Løft benet på instabil side opp fra bakken. Behold en nøytral stilling i ryggen.<br><br>Utfør øvelsen som i B.	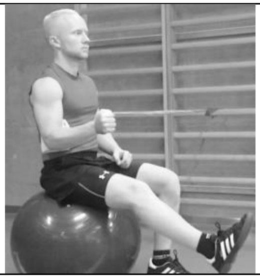	10 sek<br>3 sett<br>Daglig	Unngå å heise skulderen opp til øret.
5D	Startposisjon som i B.<br><br>Løft armen 40 grader ut fra kroppen og hold albuen i 90 grader. Tenk at armen skal forme en halv V, litt som i øvelse 1C.<br><br>Stram strikken og gjør små, rytmiske bevegelser fra side til side mens du holder skulderbladet og skulderen stabile.	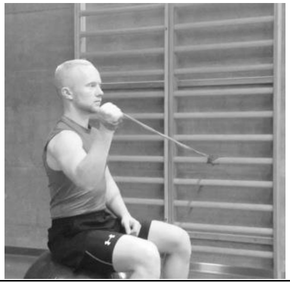	10 sek<br>3 sett<br>Daglig	Som over.
5E	Stå med beina i hoftebreddes av stand. Aktiver kjernemuskulaturen og stabiliser ryggen.<br><br>Start med armene ved siden av kroppen med 90 grader bøy i albuene. Stram strikken mellom armene.<br><br>Gjør små rytmiske bevegelser inn og ut mens du løfter armene opp over hodet og ned igjen.<br><br>Slapp av og gjenta.	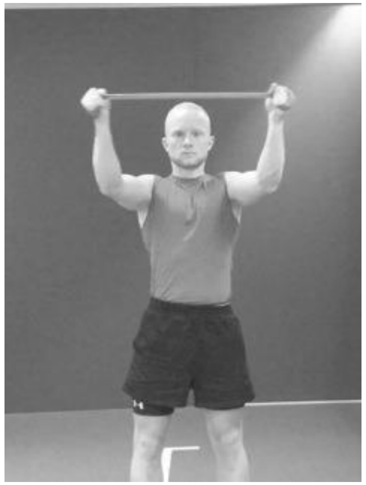	10 sek<br>3 sett<br>Daglig	Som over.

Elite nivå				
5F	Fest den ene enden av en strikk bak kroppen i skulderhøyde. Fest en flaske nært den enden av strikken som du holder i med instabil arm.<br><br>Stå med motsatt fot av instabil side fremst, knærne lett bøyd og med vekten på dem fremste foten. Ryggen er i nøytral stilling.<br><br>Plasser armen over 90 grader ut fra kroppen (kasteposisjon), med armen pekende opp mot taket.<br><br>«Sett» skulderbladet og skulderleddet. Gjennomfør små rytmiske bevegelser frem og tilbake mens du holder skulderbladet og skulderleddet stabilt. Slapp av og gjenta.	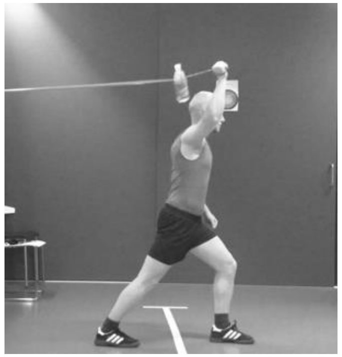	20 sek<br>3 sett<br>Tre ganger i uken	Som over.
5G	Som i F, men med vekten på én fot. Løft benet på instabil side.<br><br>Ved behov for videre progresjon:<br>Lukk øynene, løft armen høyere, eller øk motstanden fra strikken.	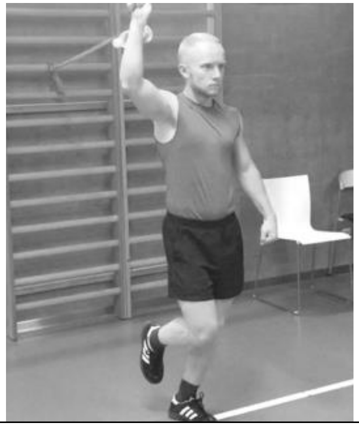	20 sek<br>3 sett<br>Tre ganger i uken	Som over.

Øvelse 6 – Leddsans i skulderleddet				
**Biomekanisk hensikt:** Å optimalisere leddsans i skulderen.				
**Praktisk hensikt:** Denne øvelsen er viktig for å forbedre leddsansen i skulderen din. Leddsans er en sans som forteller kroppen hvordan armen er posisjonert. Dette er viktig for en funksjonell skulder.				
**Generelt:** Hvis ballen faller av, la den falle. Ikke forsøk å ta i mot den. Raske bevegelser kan være uheldig for skulderen. Målet med øvelsen er å klare alle repetisjonene uten å miste ballen.				
**Grunnleggende nivå**				
**Nivå**	**Utførelse**	**Bilde**	**Dosering**	**Kommentarer**
6A	Ligg på rygg med beina bøyd.<br><br>Plasser en fitnessball i håndflatene til begge hendene med albuene bøyd. Strekk sakte ut albuene mens du balanserer ballen i hendene. Senk ballen ned igjen, slapp av og gjenta.	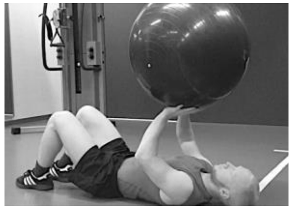 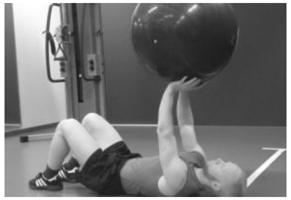	5 reps<br>2 sett<br>Daglig	
6B	Som i A, men med øynene lukket.	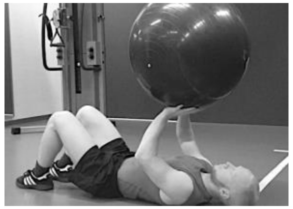 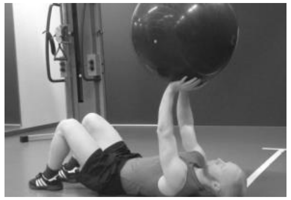	5 reps<br>2 sett<br>Daglig	

6C	Startposisjon som i A.<br><br>Plasser en fitnessball i håndflaten på instabil side med albuen bøyd. Strekk sakte ut armen mens du balanserer ballen. Hold en andre armen i nærheten av ball, i tilfelle du trenger å støtte den. Senk ned igjen, slapp av og gjenta.	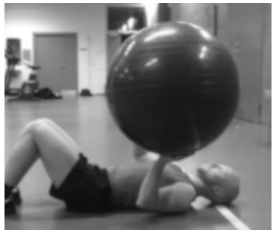 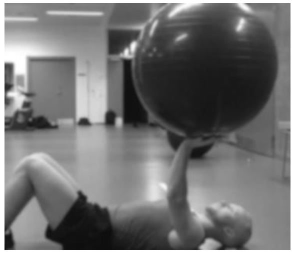	5 reps<br>2 sett<br>Daglig	
6D	Som i C.<br><br>Når armen er helt strak, fører du den rolig litt opp over hodet. Før fitnessballen inn igjen, senk rolig ned, slapp av og gjenta.	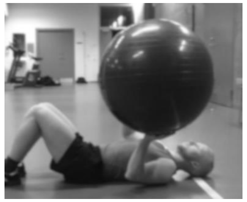 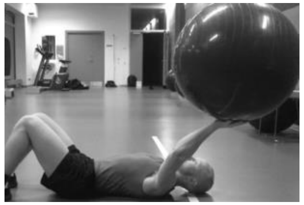	5 reps<br>2 sett<br>Daglig	
6E	Stå med den ene foten foran den andre, og motsatt ben av instabil side fremst. Knærne er lett bøyd, kroppsvekten på fremste ben, og ryggen i nøytral stilling.<br><br>Bøy albuen 90 grader på instabil side og plasser en fitnessballi håndflaten. Balanser ballen mens du strekker armen ut. Tegn en liten firkant i luften, før du tar armen tilbake. Slapp av og gjenta.	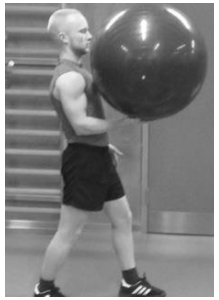 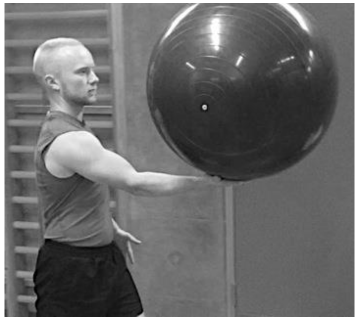	5 reps<br>2 sett<br>Daglig	

Elite nivå				
6F	Som i E, men med lukkede øyne.	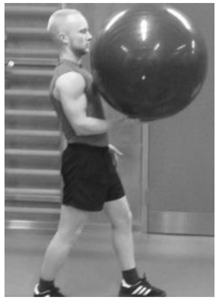 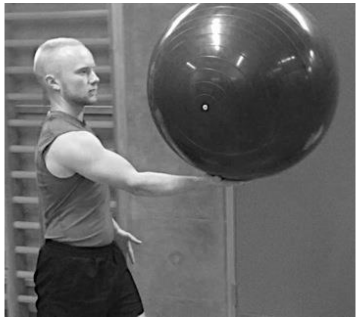	10 reps<br>2 sett<br>Tre ganger i uken	
6G	Startposisjon som i E. Gjør et utfall samtidig som du strekker ut armen og balanserer ballen. Returner rolig til startposisjon, slapp av og gjenta.<br><br>For videre progresjon:<br>Lukk øynene. Gjør en eksplosiv retur til startposisjon fra bunnen av utfallet.	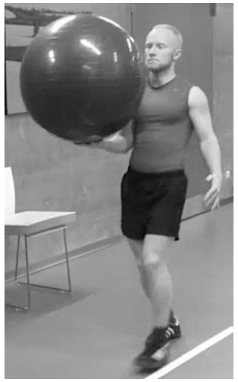 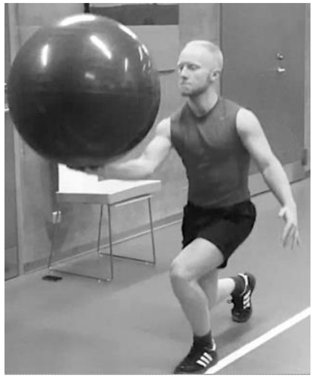	10 reps<br>2 sett<br>Tre ganger i uken	

Øvelse 7 – Leddsans i skulderleddet				
**Biomekanisk hensikt:** Å optimalisere leddsans i skulderen.				
**Praktisk hensikt:** Denne øvelsen er viktig for å forbedre leddsansen i skulderen din. Leddsans er en sans som forteller kroppen hvordan armen er posisjonert. Dette er viktig for en funksjonell skulder.				
**Generelt:**				
**Grunnleggende nivå**				
**Nivå**	**Utførelse**	**Bilde**	**Dosering**	**Kommentarer**
7A	Heng en blink på veggen i hoftehøyde. Fest en laserpeker på håndleddet på instabil side.<br><br>Stå med hoftebreddes avstand mellom føttene, og en armlengde + en fots avstand fra veggen.<br><br>«Sett» skulderbladet. Beveg armen frem med strak albue og finn midten av blinken. Lukk så øynene og tell til tre. Senk armen ned mens du holder øynene lukket og løft så opp igjen og prøv å finne midten av blinken. Åpne øynene for å se hvordan det gikk. Gjenta.	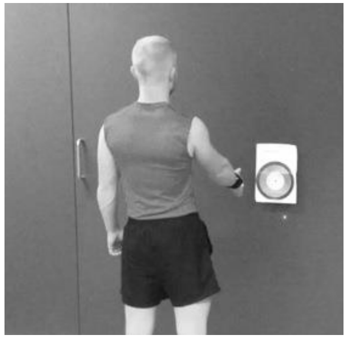	5 reps<br>2 sett<br>Daglig	Målet med øvelsen er å klare å plassere laserpekeren tilnærmet midten av blinken gjennom alle repetisjonene.<br><br>Unngå å heise skulderen opp til øret.
7B	Heng blinken på veggen i skulderhøyde. Utfør øvelsen som i A.	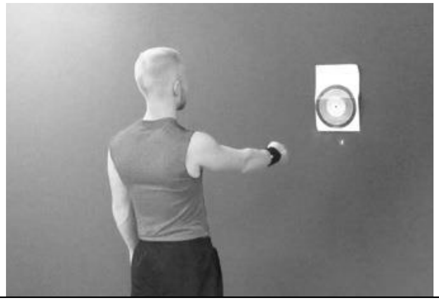	5 reps<br>2 sett<br>Daglig	Som over.

7C	Heng blinken på veggen over hodehøyde. Utfør øvelsen som i A.	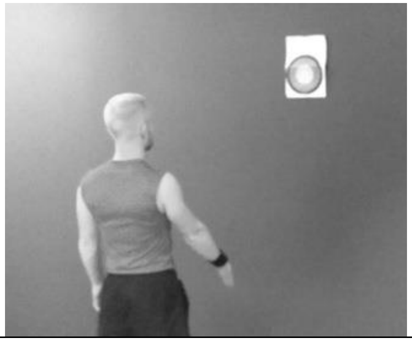	5 reps<br>2 sett<br>Daglig	Som over.
7D	Heng blinken på veggen over hodehøyde. Stå i tandemposisjon (hæl inntil tå), med benet på motsatt side av instabil side fremst. Utfør øvelsen som i A.	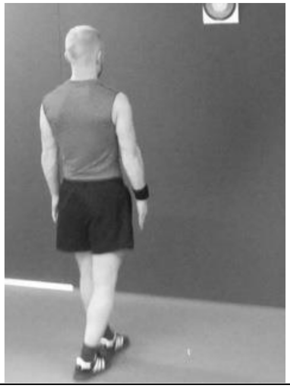	5 reps<br>2 sett<br>Daglig	Som over.

7E	Heng en blink på veggen over hodehøyde. Stå på ett ben, med benet på instabil side i luften. Utfør øvelsen som i A.	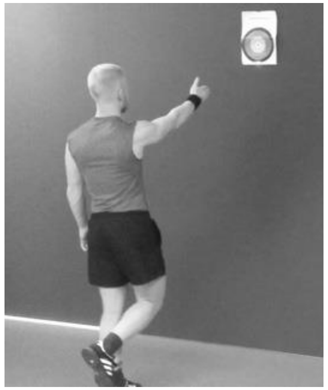	5 reps<br>2 sett<br>Daglig	Som over.
**Elite nivå**				
7F	Heng en blink på veggen over hodehøyde.<br><br>Hold en flaske med vann i hånden med laserpeker på. Løft benet på instabil side opp fra bakken og stå på en fot. Utfør øvelsen som i A.	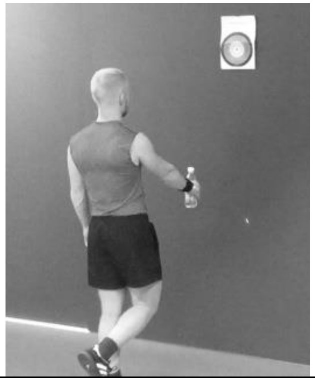	10 reps<br>2 sett<br>Tre ganger i uken	Som over.

7G	Heng en blink på veggen over hodehøyde.<br><br>Fest den ene enden av strikken under standbenet (motsatt av instabil side) og hold den andre enden i hånden på instabil side.<br><br>Utfør øvelsen som i A.<br><br>For videre progresjon:<br>Øk hastigheten på øvelsen. Heng blinken høyere.	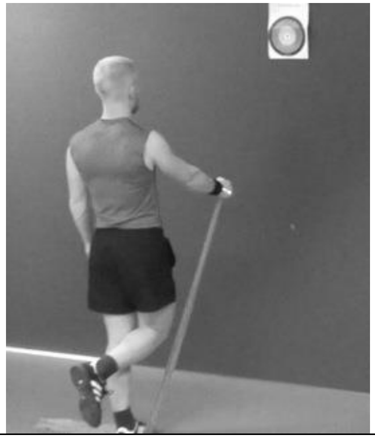	10 reps<br>2 sett<br>Tre ganger i uken	Som over.
